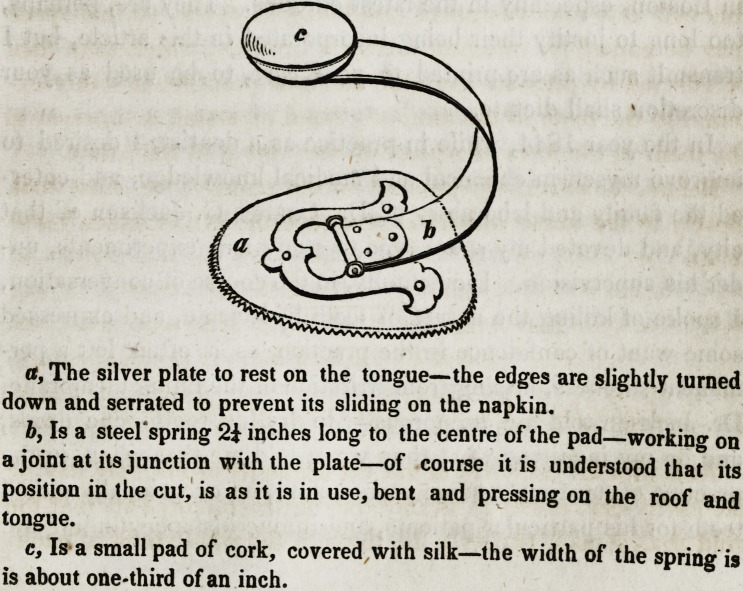# Letter from Dr. E. Townsend of Philadelphia

**Published:** 1847-10

**Authors:** E Townsend

**Affiliations:** Philadelphia


### ARTICLE VI.

### *Letter from* Dr. E. Townsend, *of Philadephia.*

All who fill teeth will admit the necessity of not only having the cavity dry, previous to introducing the filling, but that the gold shall remain perfectly dry through the whole time of packing, indeed until the whole mass is *solid.* To do this, requires that the mouth should be kept open and still, for a considerable time, (if the filling be large,) and also that the salivary ducts should be stopped as effectually as possible.

In the under jaw this is sometimes very difficult, owing to the motion of the tongue, and the activity of the sub-lingual glands.

To remedy, in some measure, the difficulty, the fixture of which the annexed cut is a representation was invented.

Its manner of use is as follows: fold a small napkin so that it shall nearly fill the space between the bicuspides, and extending posteriorly as far as the middle of the tongue, pressing it down on the sub-lingual ducts as closely as possible, then lay the broad mouth piece marked *a* upon the napkin, and passing the spring backwards, rest the pad in the roof of the mouth, the force of the spring will be sufficient to keep the napkin in its place, and to keep the mouth dry, to the end of the operation, if all the steps have been carefully taken. In some very wet mouths there is great advantage in laying on the mouths of the ducts, a small roll of soft blotting paper, and then placing the napkin over it.

*a,* The silver plate to rest on the tongue—the edges are slightly turned down and serrated to prevent its sliding on the napkin.

*b,* Is a steel spring 2¼ inches long to the centre of the pad—working on a joint at its junction with the plate—of course it is understood that its position in the cut, is as it is in use, bent and pressing on the roof and tongue.

*c,* Is a small pad of cork, covered with silk—the width of the spring is is about one-third of an inch.

## Figures and Tables

**Figure f1:**